# No evidence of multidrug-resistant *Enterobacterales* transmission between healthy companion animals and pet owners in the greater Atlanta area: a pilot study

**DOI:** 10.1128/spectrum.00503-25

**Published:** 2025-10-08

**Authors:** Wendy Cuevas-Espelid, Chiamaka U. Uzuegbunam, Jessica H. Carag, Michelle N. Hargita, Alexander M. Page, Taé C. Stallworth, Nour Makkaoui, Sarah W. Satola, Nadine G. Rouphael, Susan Sanchez, Alexandra W. Dretler

**Affiliations:** 1 Department of Infectious Diseases, University of Georgia College of Veterinary Medicine 70734, Athens, Georgia, USA; 2 Department of Medicine, Division of Infectious Diseases, Emory University School of Medicine 12239, Atlanta, Georgia, USA; 3 Atlanta Research and Education Foundation, Veterans Affairs Medical Center, Atlanta, Georgia, USA; 4 Department of Pediatrics, Children’s Hospital of Michigan, Detroit, Michigan, USA; 5 Metro Infectious Disease Specialists, Decatur, Georgia, USA; University of Kentucky, Lexington, Kentucky, USA

**Keywords:** whole-genome sequencing, companion animals, multi-drug resistance, antimicrobial resistance, selective culturing

## Abstract

Antimicrobial resistance (AMR) is a global concern affecting both animals and humans. Pets share a close bond with humans and are exposed to human-related conditions that can, in many cases, facilitate the transmission of bacteria and mobile genetic elements. This prospective observational cohort pilot study aimed to determine the prevalence of multidrug-resistant Gram-negative bacteria (MDR-GNB) colonization in healthy individuals and their companion animals (dogs and cats) in the greater Atlanta area, as well as to understand the prevalence of enteric MDR-GNB. Serial fecal samples from paired humans and their pets were collected and analyzed over a 6-month period (at 0, 2, and 6 months). Thirty-four pet owners participated, with 26 providing stool samples at all three time points. A total of 226 fecal samples were collected from owners and their pets. Seven of 26 humans and 12 of 43 animals were found to carry MDR-GNB, specifically species such as *Escherichia coli, Enterobacter ludwigii, Enterobacter hormaechei,* and *Citrobacter pasteurii*. Whole-genome sequencing revealed nine different resistance genes in *E. coli* isolates from pets and eight from humans, six different plasmid replicons, and all were located in four different phylogroups. Phylogenetic analysis indicates species-specific clustering based on host. Our results demonstrate that while MDR Enterobacterales were present in both humans and their pets in this Atlanta population, there was no evidence of bacterial transmission between pets and their owners during the study period. This finding contradicts previous similar studies that have shown transfer of MDR bacteria. However, it aligns with research that suggests bacterial colonization depends on the strain and the host.

**IMPORTANCE:**

Antimicrobial resistance in animals, particularly pets, may serve as a potential source of antimicrobial resistance. However, a definitive pathway for the transmission of clonal bacteria or horizontal gene transfer between humans and their pets has not yet been identified. This pilot study aimed to assess the risk of multidrug-resistant (MDR) Enterobacterales transmission between healthy humans and their companion animals (dogs and cats) in the greater Atlanta area. Additionally, it sought to explore any association between MDR bacterial colonization and transmission within participating households. Despite the lack of a fully defined method of transmission, our findings demonstrated that while MDR *Enterobacterales* were present in both humans and their pets in this Atlanta population, there was no evidence of bacterial transmission between pets and their owners during the study period.

## INTRODUCTION

Antimicrobial-resistant (AMR) bacteria are a serious global threat to human and animal health as they are the cause of high morbidity and mortality in many species worldwide (1–3). Even when naturally present in the intestinal microbiota of healthy individuals, multidrug-resistant Enterobacterales (MDR-E) are a significant contributor to both veterinary (4–12) and human hospital-acquired infections, leading to increased morbidity, mortality, and escalating healthcare costs (13–16). Tackling antimicrobial resistance will require a coordinated, comprehensive approach involving medical, veterinary, ecology, and public health experts (17–19).

Most of the AMR pathogenic bacteria species are in the order *Enterobacterales*, which contains seven families, with the Family *Enterobacteriaceae* having one of the most significant impacts on the health of people and animals globally (20). The major bacterial pathogens in this family include *Escherichia coli*, *Salmonella spp*., *Shigella spp.,* and *Yersinia enterocolitica* (21–24). Strains of extended-spectrum beta-lactamase-producing *Enterobacterales* (ESBL-E) and carbapenem-resistant *Enterobacterales* (CRE) have been identified as both intestinal commensal and pathogenic bacteria in multiple animal populations, including cattle, pigs, and chickens (25–28). Wild birds and the environment, such as freshwater and soil, can contribute to the spread of MDR-E, raising further concern about transmission (29–31). Acquisition of AMR bacteria by people is believed to occur through multiple transmission routes, such as handling and eating contaminated food, direct contact with food-producing animals or animal waste (28), or through exposure to hospital or community-acquired infections (32, 33). However, there is current research indicating that transmission is not straightforward as previously thought (34, 35). Commensal reservoirs of ESBL bacteria in the environment have seen a sharp rise recently, driven by the transmission of ESBL-E between human and animal populations, which can occur through various direct and indirect transmission pathways (36).

Virulence and resistance genes can be shared between bacteria primarily through horizontal gene transfer (HGT), with mobile genetic elements serving as the main vehicles for DNA dissemination (37). Additional mechanisms, such as compensatory or adaptive mutations, can also contribute to the maintenance of host defense systems, which include virulence and resistance genes, as they adapt and persist in competitive and challenging environments, including new ecological niches (38, 39).

Little is known about the prevalence of AMR in companion animals and their potential as reservoirs of resistant bacteria. This is a significant issue, as over 45% of US households have a dog or cat at home (40). Studies in companion animals have revealed evidence of stool colonization with ESBL-E in otherwise healthy dogs and cats (8, 41–44). Infections caused by MDR Enterobacterales (MDR-E) in companion animals are increasingly diagnosed, often following treatment failure and recurring infections in areas like the urinary tract, ears, skin, surgical sites, and respiratory tract (45–48). Furthermore, evidence of household transfer of ESBL-E between humans and their pet dogs has also been reported, suggesting that transmission of resistant pathogens between companion animals and their owners seems to occur (4, 7, 9, 49, 50). The role of companion animals as reservoirs of resistant bacterial infections may be a critical aspect of infection control (51).

The human-animal bond is distinctive because the anthropomorphic environments we have created for pets lead us to instinctively believe that resistant bacteria and genes can be readily exchanged between humans and animals. Nevertheless, the effectiveness and exact transmission mechanisms between humans and their companion animals are yet to be definitively determined, and transmission could be direct or indirect from similar exposures to a shared food or environmental source (52). This pilot study set out to determine the prevalence of MDR-GNB colonization in healthy humans and their companion animals (dogs and cats) in the greater Atlanta area and to further examine whether there is a transmission between MDR bacterial colonization in healthy humans and their pets. We hypothesize that there will be transmission of MDR bacteria between pet owners and their pets and/or vice versa. This unique and novel study prospectively obtained serial fecal samples from matched humans and companion animals. The samples were analyzed using selective media to isolate MDR Gram-negative bacteria (MDR-GNB), specifically those resistant to at least ESBLs, ceftriaxone, and meropenem. The isolates were characterized through whole-genome sequencing (WGS) for the presence of MDR-E to better understand the colonization and transmission of this group of enteric bacteria in households in the United States and the potential health risk factor. An integrated One Health approach is required to understand the intricacies of the transfer of MDR between owners and their pets.

## RESULTS

### Sample metadata survey breakdown and prevalence of fecal GNB in pets and their owners

A total of 34 pet owners were enrolled in this study, of which 26 (n_women_ = 22, n_men_ = 4) provided a stool sample at all three time points. Out of this sample size, 88.5% (*n* = 23) of the participants were white, 7.7% (*n* = 2) were African American, and 3.8% (*n* = 1) were American Indian. The ages of the participants ranged from 25 to 69 years (median 37 years, mean 43 years). Over the study period, 7 (27%) out of the 26 participants had at least one stool sample test positive for GNB grown on selective media. Stool samples were available at all three time points from 43 pets (n_dogs_ = 28, n_cats_ = 15). Twenty-eight (28/43, 65%) pets were male. The age of the pets ranged from 4 months to 13 years (median 7 years, mean of 7 years). The pets in the study had variable sleeping arrangements and diets, with 67% (28/43) sleeping in the same bed as their owners, 95% (41/43) consumed dry food regularly, 40% (17/43) consumed wet food regularly, and 9% (4/43) consumed raw food regularly. Most pets were spayed or neutered (94%, 40/43), and 98% (42/43) lived primarily indoors. One-third of the pets (33%, 14/43) had a history of chronic veterinary problems requiring more frequent veterinary visits or chronic medications. Additionally, three participating households had non-dog/cat pets (sun conure, guinea pigs, and bearded dragons), which were not included in this study. Fifty-four pets were initially enrolled in the study, and 11 animals were excluded from participation due to the reasons previously stated. Overall, 15 (35% 15/43) pets had at least one stool sample test positive for GNB at any point in time during the study. Table 1 shows the descriptive data and prevalence of GNB colonization among study participants and their pets. No significant association was found between pets and human subjects that were positive for GNB and positive for MDR-E (*P* = 0.19). Odds ratios were calculated (Table 2) to interpret the risk of GNB colonization by the descriptive characteristics laid out in Table 1. None of the odds ratios calculated were significant at the *P* = 0.05 level.

**TABLE 1:** Population sample breakdown of enrolled participants

Variables		Total pets	Prevalence of MDR GNB Isolation over all three visits or at time 0, 2, or 6 months
Owner’s age (years)	25-–34	12	17%
	35–44	4	25%
	45–54	1	100%
	55–64	8	38%
	>64	1	0%
Owner’s sex	Male	4	0%
	Female	22	32%
Owner’s race	White	23	30%
	African American	2	0%
	American Indian/Alaska Native	1	0%
Pet type	Cat	15	20%
	Dog	28	32%
Pet sex	Male	28	29%
	Female	15	27%

**TABLE 2:** Odds ratios based on descriptive characteristics

Characteristics	Colonization OR (95% CI) [*P*-value]^*a*^
Male subject	0.23 (0.01, 4.84) [0.34]
Female subject	4.35 (0.21, 91.87) [0.34]
Cat	0.53 (0.12, 2.35) [0.40]
Dog	1.89 (0.43, 8.43) [0.40]
Male pet	1.10 (0.27, 4.50) [0.89]
Female pet	0.91 (0.22, 3.72) [0.89]

^*a*^ The established level of significance is a *P*-value < 0.05. A 95% confidence interval was used to calculate the odds ratios.

### Selective lab-late ID, matrix-assisted laser desorption/ionization time-of-flight, and WGS GNB species identification

A total of 226 fecal samples were obtained from 34 pet owners and their pets. Of these, 33 (15%) were positive for growth on Chrome ESBL agar, and only six yielded growth on Chrome MacConkey CRE. Laboratory phenotypic identification through lab plate ID and matrix-assisted laser desorption/ionization time-of-flight (MALDI-TOF) revealed 29 *E. coli*, five *Klebsiella pneumoniae*, one *Enterobacter cloacae/asburiae*, and one *Pseudomonas aeruginosa.* The WGS analysis of the 22 selected strains corroborated the phenotypic identification by a combination of lab plate ID and MALDI-TOF in the majority of the cases (Data S5). Both methods determined that the most common isolated organism in both animals and pet owners was *E. coli* (*n* = 18 via lab plate ID, *n* = 19 via WGS)*.* The other isolates were identified as *Enterobacter hormaechei* found in a pet and *Citrobacter pasteurii* and *Enterobacter ludwigii,* both found in two humans (Table 3). In total, 21 isolates showed phenotypic antimicrobial resistance and/or carried resistance genes via STREK PCR. Eight isolates presented multidrug resistance via disc diffusion that included 17 different antibiotics. Due to shortages of laboratory consumables during the COVID-19 pandemic, antimicrobial susceptibility testing via disc diffusion was only completed for 13 isolates (Table 3).

**TABLE 3:** AMR genes found in the chromosome or plasmid and predicted phenotype from humans (>25 years old) and animals identified using WGS^*a*^^,^^*b*^

Isolate ID	STRECK PCR results	AMR gene (WGS)	Phenotypic resistance based on DD	Predicted resistance phenotype based on antibiotic class
EC_301T2_P1	*ampC*	*blaCMY-2*	NT	Penicillins, cephalosporins
EC_301T2_P2	*B-Lactamase, ampC*	*blaCMY-2* IncFII (26) IncFII(pHN7A8)	AMP, AZ, CE, CX, CZ, CT	Penicillins, cephalosporins
EC_301T6_P2	*B-Lactamase, ampC*	*blaCMY-2* IncFII (26)	NT	Penicillins, cephalosporins
EN_302T2_H1	*B-Lactamase, ampC*	*blaACT-12* *blaNMC-A*	AMP, CE, CX, E, M, P	Penicillins, cephalosporins, phosphonics
EN_302T2_P3	*B-Lactamase, ampC*	*blaACT-16*	CE, CX	Penicillins, cephalosporins
EC_303T6_H2	*B-Lactamase, ampC*	*blaCTX-M-15* IncFII(pRSB107)	NT	Penicillins, cephalosporins, quinolones
EC_303T2_H2	*B-Lactamase, ampC*	*blaCTX-M-15* IncFII(pRSB107)	AZ, CE, CF, CZ, CT, L	Penicillins, cephalosporins, quinolones
EC_303T0_H2	*B-Lactamase, ampC*	*blaCTX-M-15* IncFII(pRSB107)	AMP(I), AZ, CE, CZ, CT, L	Penicillins, cephalosporins, quinolones
CF_304T6_H3		none	NT	Sensitive
EC_304T2_P4	*B-lactamase*	*aadA1*(P) *& aadA2cmlA*, *dfrA12*, *sul3* IncR *blaCMY-2* IncI1-I(Alpha) *blaTEM-1B, gyrA (D87N), gyrA (S83L), parC(S80I), tet(A)*	AMP, CE, CX(I), CT, L, T, TR	Aminoglycosides, penicillins, cephalosporins, amphenicol, sulfonamides, quinolones, tetracycline
EC_304T2_P5	*B-Lactamase, ampC*	*blaCMY-2*(P) IncFII(pHN7A8)	AMP, CE, CX, CZ, CT	Penicillins, cephalosporins
EC_305T2_P6	*B-Lactamase, ampC*	*ant(2'')-Ia, blaCMY-2, cmlA1, qacE blaTEM-1B* (IncI1-I(Alpha))	P, C, A(I)	Aminoglycosides, penicillins, cephalosporins, amphenicol, quaternary ammonium compounds
EC_311T0_H4	*B-Lactamase, ampC*	*blaCTX-M-27* *blaTEM-1B* (IncFII (26))	NT	Penicillins, cephalosporins
EC_311T2_H4	*B-Lactamase, ampC*	*blaCTX-M-27* *blaTEM-1B* (IncFII (26))	NT	Penicillins, cephalosporins
EC_311T2_P7	*B-Lactamase, ampC*	*blaCMY-2* IncFII, IncFII(pHN7A8), IncI1-I(Alpha)	NT	Penicillins, cephalosporins
EC_313T0_H5	*B-Lactamase, ampC*	*aph(3'')-Ib, aph (6)-Id , blaCTX-M-14, gyrA (D87Y), gyrA (S83L), parC (S80I), sul2*	NT	Aminoglycosides, penicillins, cephalosporins, quinolones, sulfonamides
EC_313T2_H5	*B-Lactamase, ampC*	*aph(3'')-Ib, aph (6)-Id, blaCTX-M-14, gyrA (D87Y), gyrA (S83L), parC (S80I), sul2*	NT	Aminoglycosides, penicillins, cephalosporins, quinolones, sulfonamides
EC_313T0_P8	*B-Lactamase, ampC*	*blaCMY-2* IncFII	NT	Penicillins, cephalosporins
EC_322T2_H6	*B-Lactamase, ampC*	*aac (3)-IId, aadA5, dfrA17, mph(A), qacE, sul1* IncFII (26) *[aac (3)-IId, aph(3'')-Ib, aph (6)-Id, blaCTX-M-15*, *blaTEM-1B, sul2, tet(A)* (IncI1-I(Alpha))] *gyrA (S83L)*	NT	Aminoglycosides, penicillins, cephalosporins, sulfonamides, quinolones, macrolides, quaternary ammonium compounds, tetracycline
EC_322T0_P9	*B-Lactamase, ampC*	none	NT	Sensitive
EC_322T0_H6	*B-Lactamase, ampC*	*aac (3)-IId, aadA5, aph(3'')-Ib, aph (6)-Id, blaCTX-M-15, sul2, tet(A)* IncI1-I(Alpha) *blaTEM-1B, dfrA17, gyrA(S83L), mph(A), qacE, sul1*	NT	Aminoglycosides, penicillins, cephalosporins, quinolones, macrolides, quaternary ammonium compounds, sulfonamides, tetracycline

^*a*^ Host spectrum of identified resistance genes and predicted phenotypic by ResFinder are listed by class of antibiotic with each isolate having one or more of the following classes; penicillins (ampicillin, amoxicillin/clavulanic acid), cephalosporins (cefoxitin, ceftriaxone), quinolones (ciprofloxacin I/R, nalidixic acid), sulfonamides (sulfisoxazole, trimethoprim), aminoglycosides (gentamicin, streptomycin, kanamycin), phosphonic (fosfomycin), macrolides (erythromycin, azithromycin), quaternary ammonium compounds (qacE_1_X68232), amphenicol (chloramphenicol). Results of disc diffusion and PCR are included in Data S5. Antibiotics used in disc diffusion (DD) are abbreviated as follows; amikacin (AM), ampicillin (AMP), aztreonam (AZ), cefazolin (CE), cefepime (CF), cefoxitin (CX), ceftazidime (CZ), ceftriaxone (CT), ertapenem (E), gentamicin (G), levo (L), meropenem (M), pip-tazo (P), tetracycline (T), tigecycline (TG), tobramycin (TO), and trim-sulfa (TR). (I) indicates intermediate resistance and (NT) indicates not tested. (Genes identified were found within the core genome, or if the plasmid name is present below genes, this indicates that the AMR gene was located within the plasmid).

^*b*^ Isolate identification in this manuscript figures and tables is as follows: For this paper, the first two letters denote genus and species (EC: *E. coli*, EN: *Enterobacter ludwigii/hormaechei/cloacae*, CF: *Citrobacter freundii*, followed by the cohort number. Then the time of collection is denoted as T0, T2, or T6, indicating sample collection at baseline, 2 months, or 6 months. Then the last part of the number denotes the host, H denoting bacterial isolates obtained from human participants (e.g., EC_303T0_H2), and P denoting pet isolates with further numerical identification if more than one pet per household by the addition of a consecutive number (e.g., EC_304T2_P4 and EC_304T2_P5).

### Genomic characterization of AMR ***E. coli* isolated from pet owners and their animals**

**Fig 1:**
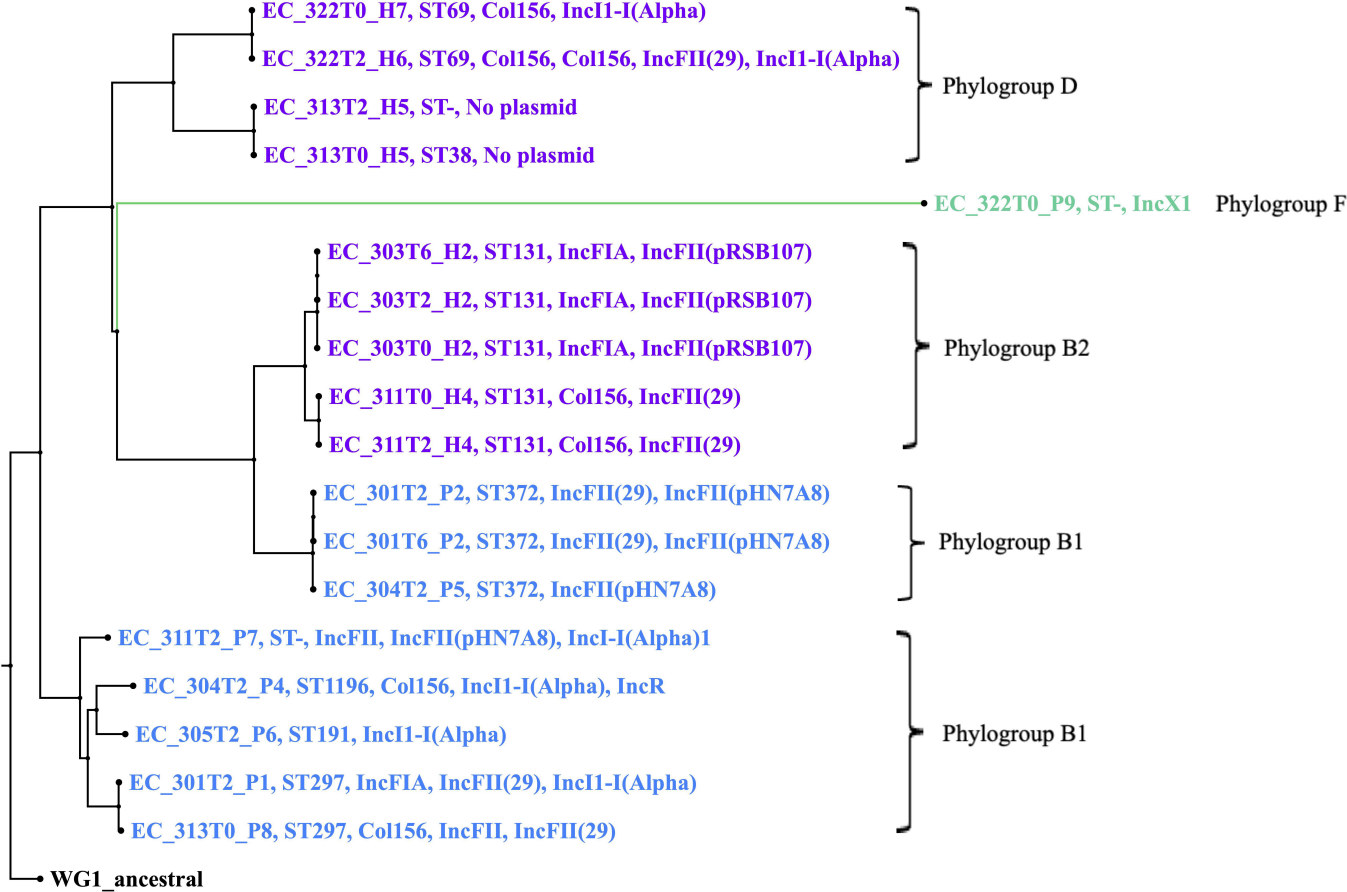
Core genome Maximum-Likelihood phylogenetic analysis, including all *E. coli* isolates identified, including those that did not have a complete sampling time period. Pet *E. coli* is indicated in blue, and human *E. coli* is in purple. Singleton EC_322T0_P9 appears in green. Strain type (ST), plasmid type, and *E. coli* phylogroup have also been included.

The *E. coli* isolates were further classified using a Multi-Locus Sequence Typing (MLST) analysis that grouped them by sequence type (ST) based on seven housekeeping genes: *adk, fumC, gyrB, icd, mdh, purA,* and *recA*. Among the 19 *E. coli* isolates, nine different STs were identified. *E. coli* MLST results for pets included ST1196, ST1131, and ST191 in one pet each, and ST372 observed in three pets (Table 4). In pet owners, ST38 and ST895 were identified in one human each, ST69 and ST297 were identified in two humans each, and extra-intestinal pathogenic *E. coli* (ExPEC) ST131 was identified in five different human isolates. Two humans and two pets carried *E. coli* for which a ST could not be assigned. Additionally, the *in silico E. coli* phylotyping system developed by Clermont et al. in 2000 (53) placed the 19 *E. coli* of this study in phylogroup B1, B2, D, or F. Both humans and animals carried *E. coli* strains from phylogroup B1, but most *E. coli* strains isolated from pets (8 dogs and 1 cat) belonged to phylogroup B1. Phylogroup B2 is the most prevalent among our sample set. It has only been found in pet owners, followed by phylogroup D. Sample EC_322T0_P9 is the only isolate categorized in phylogroup F, a recent *E. coli* phylogroup. A core genome phylogenetic analysis that included all recovered *E. coli* isolates encompassing all recovered *E. coli* isolates at all time points is visualized in Fig. 1. An ancestral *E. coli* K-12 (NCBI accession no. SAMN29011448) was included as an outgroup. The clusters of isolates suggest a host species-specific nature, with three main clades and eight subclades that coincide with Clermont’s phylogroups and clearly segregate the *E. coli* isolates by host (Fig. 1). In this analysis, isolate EC_322T0_P9 again appeared as a singleton, and further phylogenetic analyses showed that it was more closely related to the so-called *E. coli* “cryptic clades” (54) than to any of the other isolates of this study (Data S2 and S3).

**TABLE 4:** MLST of *E. coli* isolates^*a*^^,^^*b*^

Isolate	ST
EC_301T2_P1	297
EC_301T2_P2	372
EC_301T6_P2	372
EN_302T2_H1	895
EN_302T2_P3	1131
EC_303T6_H2	131
EC_303T2_H2	131
EC_303T0_H2	131
CF_304T6_H3	–^*c*^
EC_304T2_P4	1196
EC_304T2_P5	372
EC_305T2_P6	191
EC_311T0_H4	131
EC_311T2_H4	131
EC_311T2_P7	–
EC_313T0_H5	38
EC_313T2_H5	–
EC_313T0_P8	297
EC_322T2_H6	69
EC_322T0_P9	–
EC_322T0_H7	69

^*a*^ Sequence Type (ST) results obtained from MLST software.

^*b*^ EN, *Enterobacter ludwigii/hormaechei/cloacae*; EC, *Escherichia coli*; CF, *Citrobacter pasteurii*.

^*c*^ “–” indicates that the sequence type was not determined by the software.

There were nine distinct resistance genes found in pets and eight in humans, with 15 different isolates carrying the resistance gene on a plasmid. Only resistance AMR gene *bla-TEM-1B* and three point mutations, two in *gyrA* and one in *parC*, were carried by bacteria isolated from both pets and people, with bla-TEM-1B located on both the chromosome and within a plasmid (Table 3). Specifically, chromosomal point mutations gyrA (D87N), gyrA (S83L), and parC (S80I) were shared by pet *E. coli* EC_304T2_P4 and human *E. coli* EC_313T0_H5, and point mutation gyrA (S83L) was identified in human *E. coli* EC_322T2_H2 and pet EC_322T2_H6 (Table 3).

**Fig 2:**
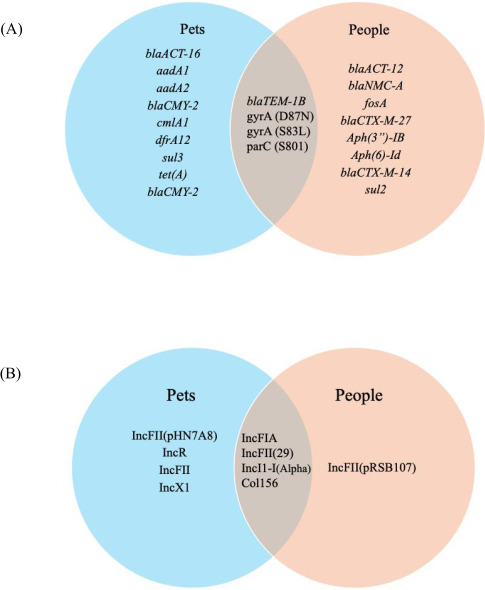
Venn diagrams depicting shared and non-shared AMR genes (**A**) and plasmids (**B**). Four plasmids were identified distinctly in pet isolates and one (1) for the human isolate. Four plasmids are identified in pets and humans, all from separate households.

In total, six different plasmid replicon types were identified. Plasmid types found exclusively in pets included IncFII(pHN7A8) found in three pets, and IncFII, IncR, and IncX1, each of them carried by a different animal. Plasmids found singularly in humans only included type IncFII(pRSB107). There were no plasmids shared between animals and their owners. However, several plasmid types were found in pets and humans that did not share a household, including IncFIA, IncFII (26), IncI1-I(Alpha), and Col156 (Table 2; Fig. 2; Data S4).

## DISCUSSION

Current literature does not detail a clear path for the dissemination of clonal bacteria and/or HGT between humans and their pets. Further research is needed on the dynamics of transmission and acquisition of MDR-E between humans and pets in the home to inform and tailor infection control practices. This pilot study examined the prevalence of GNB colonization in humans and pets in the greater Atlanta area, revealing MDR-E stool colonization in healthy, asymptomatic individuals and pets that had no recent history of antibiotic use. Specifically, in this cohort, 27% (7 out of 26) of pet owners (22 females and 4 males), 29% (8 out of 28) of dogs, and 7% (1 out of 15) of cats contained MDR-E in their feces. Similar studies have shown AMR carriage in humans and animals (7, 55). In one study, 171 healthy pets (cats and dogs) were evaluated to determine the rate of fecal carriage of *E. coli* ESBL strains, and it was found that 11.7% were carrying ESBL-producing *E. coli* (44). Dogs are more likely to carry ESBL-producing *E. coli* than other companion animals, presenting a greater risk as ESBL reservoirs for opportunistic infections (56, 57). This can have profound outcomes, as the presence of these MDR potential pathogens might increase the risk for opportunistic infections (57). Surprisingly, we did not find an association between MDR-*Enterobacterales*-carrier pets and MDR-E-positive owners (55, 58). Under our selective collection method, *E. coli* was the most prevalent bacterium isolated from feces. Furthermore, we were able to detect the same *E. coli* strains over time from both pets and humans. This could indicate a stable microbiome for the host for the duration of the study. Other species, such as *E. hormaechai*, *E. ludwigii,* and *C. pasteurii,* appeared sporadically despite being natural commensals of the animal and human gut microbiota with the potential to cause nosocomial infections in humans (59–61).

There were discrepancies between MALDI-TOF and the WGS species identification methods used in our study. In this study, species identification was performed using MALDI-TOF (62), which, in some cases, yielded contradictory results. WGS confirmed species identification. Although both techniques agreed on the identification of the majority of bacterial species, MALDI-TOF misclassified one *E. ludwigii* sample as *Citrobacter* spp (63, 64). The advances in WGS and the decrease in its price have encouraged many veterinary diagnostic labs to explore the idea of using this technology for microbial identification (65). This supports previous findings that consider WGS to be more accurate than MALDI-TOF for identifying bacteria, particularly when distinguishing closely related species or detecting novel or rare pathogens (66).

Phenotypic antimicrobial susceptibility testing methods are reasonably accurate, simple to perform, inexpensive, and continue to be valuable and relevant in clinical laboratories. Nevertheless, definitive results may not be received in a timely manner to initiate treatment for a patient, despite being referred to as the “gold standard” (67, 68). This study was conducted during the height of COVID-19, which hindered the completion of phenotypic susceptibility testing. Despite this, the eight MDR isolates matched with the genotypic result from WGS analysis with regard to the antibiotic classes penicillins and cephalosporins. However, there was one isolate that was phenotypically resistant to meropenem but did not have the corresponding genotype as determined by WGS. This discrepancy has been noted in other studies (69). Despite this one-off result, WGS still offers more precise results for identification, control of bacterial infections, and epidemiological applications (70).

*E. coli* became the focus of our study as it was the only MDR-E species consistently found in the donor feces. Phylogenetic analysis revealed distinct clades that grouped humans and pets separately, highlighting the species-specific nature of the isolates. We hypothesize that the species-specific clustering occurred between humans and their pets due to differences in their microbiome and diet.

Our genotyping study revealed that most of the isolated *E. coli* in pets and owners belonged to phylogroups B1, B2, and D. Phylogroup B2 is the most prevalent among our sample set and predominantly found in humans, which is consistent with previous work (71). In fact, the most common human ExPEC strain worldwide is ST131 and was identified in our sample set (72). The most common STs in the pet *E. coli* were ST372 and ST297. Similar to our study, other research on *E. coli* in dogs has predominantly identified strains belonging to known phylogroups B1, which included STs 191, 297, 372, and 1196 (73–75). These strains are part of the normal canine microbiota, and they are also considered opportunistic pathogens, commonly associated with extraintestinal infections in humans and animals (56, 72).

Isolate EC_322T0_P9 was found as a singleton in our tree, sharing high genetic similarity with *E. coli* cryptic clades*. Escherichia* cryptic clades are unique lineages within the *Escherichia* genus that share a close relationship with typical *E. coli* strains but differ in their genetic and phenotypic characteristics (54). This single cryptic *E. coli* is of public health relevance since it is indistinguishable from *E. coli* via standard laboratory procedures. Cryptic clades can thrive within the environment and serve as commensal and/or pathogenic bacteria; hence, research has primarily focused on the presence of these cryptic clades in various environments and hosts such as livestock and avian species (76). However, their association with canine hosts has not been well understood (54). Pet 322T0_P9 consumed a raw diet. Hence, we hypothesized that this could be the introduction route of this strain to the animal. Raw diets continue to be controversial as the zoonotic component and danger to human health from a raw meat-based diet concern public health officials (77, 78). Strong efforts to educate clients and improve awareness are ongoing by veterinarians (79, 80).

The relationship and bond between humans and animals can be deeply affectionate, friendly, and intimate. While previous studies have demonstrated that companion animals possess resistant genes and bacteria shared with humans, there might be alternative routes of exposure that enable colonization, such as raw diets (81). Although other *Enterobacterales* species were detected, only single isolates of each were found, preventing meaningful comparisons; hence, *E. coli* became the only bacterial species further characterized. This pilot study did not show transmission of MDR *E. coli* between pet owners and their animals. People from different households were colonized with different strains of *E. coli*. However, there were shared AMR genes and plasmids in the strains present in the different households, suggesting a different source of exposure and/or genes shared in plasmids via HGT. This result differs from other studies (4, 7, 9).

There were some limitations to the study, including the use of Illumina-generated short read sequences, which offer limited plasmid analysis due to the nature of their size. Subsequently, complete plasmid assembly is frequently not possible with short reads (82). Redundancy is a common situation in plasmid genomes and can introduce assembly ambiguity and fragment assemblies (83, 84). Long-read sequencing is a more suitable method for identifying plasmids, as it can accurately resolve repetitive regions, detect inversions, and uncover other large-scale structural anomalies within the sequence (85). Some plasmids contain multiple replicons within the same sequence, creating a limited resolution for epidemiological purposes (86, 87).

Few studies have longitudinally examined the transmission of MDR bacteria between humans and their companion animals (50). The relatively small sample size limited the power for further analysis, and the convenience sampling method, alongside insufficient diversity in geography, race, and health status, affected the generalizability of the results. Also, there was a proportionately higher participation rate for women pet owners, which makes us question whether the interpersonal relationship is different with male pet owners.

The MDR *E. coli* identified in the people and their pets’ normal microbiome maintained stable colonization throughout the study, as evidenced by consistent characterization results over the study time. Our study also raised questions about whether the microbiomes of humans and companion animals offer a protective barrier against infections and pathogenic bacteria, with potential dietary influences (88). Furthermore, different *E. coli* strains colonize only humans or pets. Different microbiomes may influence bacterial presence and the potential transfer (88) of resistance. Companion animals have a very different nutritional source than their owners, and people have different diets, all of which have clear implications for their microbiome (89–93). These variations in their microbiome determine bacterial (*Enterobacterales, E. coli*) colonization, shedding, and AMR transfer, which could be the case in our study (91, 92).

One Health implications regarding pet ownership and raw diets reinforce the unresolved questions and a deeper understanding of the underlying forces that AMR genes and plasmids play in the microbiome of humans and animals (94). Additionally, outdoor environmental influences that significantly contribute to the exposure of multiple hosts to MDR bacteria cannot be ignored. The diversity of AMR genes, plasmids, STs, and phylogroups that were encountered in this study has implications for biodiversity conservation, agriculture, and public health (95).

## MATERIALS AND METHODS

### Study design

Participants were recruited from the community in the greater Atlanta area between July 2018 and December 2019. They were eligible for screening if they were over 18 years of age, in general good health, at a low risk for colonization with MDR bacteria, and had at least one companion animal (cat or dog) in the household. Participants were excluded if they had a history of any MDR bacterial infection, had traveled to an endemic ESBL region (including Southeast Asia, the Eastern Mediterranean, or the Western Pacific) (36, 96, 97) within the last year, had been hospitalized for more than 24 hours in the previous year (excluding childbirth), or had taken any systemic antibiotics during the previous 6 months.

Finally, participants were also excluded if any other human household member had been hospitalized for more than 24 hours in the last year or had traveled to an endemic ESBL region (98) within the previous year. Participants were also excluded if any of the companion animals in the household had a history of MDR bacterial infections, had been prescribed systemic antibiotics in the last 6 months, or had been hospitalized in a veterinary hospital for more than 24 hours in the last year.

**Fig 3:**
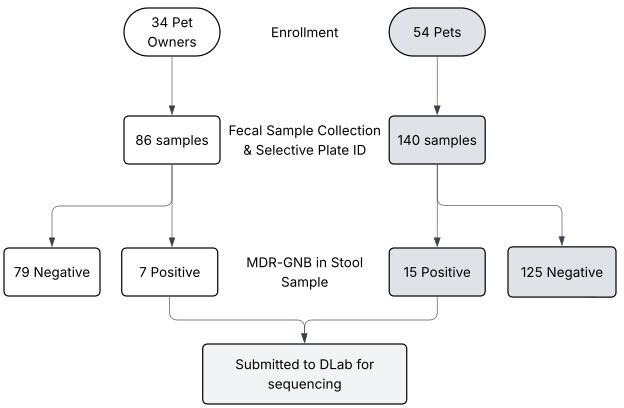
Study workflow with the final number of community recruitment and stool samples submitted for WGS.

During the initial enrollment visit, detailed demographic data, medical history, medications, and social history were collected for both the participants and their pet(s). Targeted veterinary history of companion animal(s), including signalment (age, sex), breed of the animal(s), date of the most recent veterinarian visits or veterinary hospitalizations in the last year, and any medications in the previous 30 days, was recorded. A detailed social history regarding dog and/or cat ownership was also performed. Specifically, the details of cohabitation between the participant and the companion were reviewed with a focus on obtaining the details of where the animal(s) reside and spend the majority of their time (indoor vs. outdoor), where the animal(s) sleep (i.e., in crate vs. in bed with the participant), the animal’s primary diet (i.e., raw commercial pet food vs. non-raw commercial pet food vs. shared human food), and who in the household is the primary caretaker of the companion animal(s) (subject vs. another member of household). All additional pets (not cats or dogs) in the household were also documented. Follow-up visits and stool collection were conducted at 2 and 6 months. Participant, companion animal medical, and social history were reviewed and updated with a specific focus on risk factors for MDR colonization, including doctor or veterinary visits, hospitalizations, antibiotic use, and travel. See Fig. 3 for the study workflow and sampling strategy.

### Stool collection and bacterial culture

All participants were provided written instructions entitled “Stool collection Instructions for ARCA Study” on how to collect stool samples at home and submit them to the laboratory at the time of their visit (Data S1). Using a 10 µL inoculating loop, one loopful of stool was obtained and placed in brain heart infusion (BHI) broth (Becton Dickinson BBL) at 37°C for overnight enrichment. A second loopful of raw stool was placed in a cryogenic vial and frozen at −80°C as a backup. Agar plates were purchased pre-poured from the manufacturers as indicated (Becton Dickinson and Company, Sparks, MD, and Hardy Diagnostics, Santa Maria, CA) and stored at 4°Celsius until use. Then, they were taken out and inoculated with a fecal sample, followed by incubation at 35^o^ ± 2°C overnight. On day 2, the enriched broth was plated onto MacConkey agar with a carbapenem disk (10 μg meropenem) and a cephalosporin disk (30 μg ceftriaxone) (Becton Dickinson and Company, Sparks, MD) and onto HardyChrom ESBL agar (Hardy Diagnostics, Santa Maria, CA) according to the manufacturer’s instructions (99, 100). Well-isolated colonies recovered from the different media were grown on BHI broth and diluted in 15% glycerol and peptone water for storage at −80°C for later DNA extraction.

### Isolate molecular characterization

If growth was only observed on the ESBL plate or within the zone of inhibition on the MacConkey plates, then qPCR was performed using the protocol referenced (101). The preparation of primers and probes for the detection of β-lactamases and *ampC* genes was conducted per the manufacturer’s instructions using the β-Lactamase and *ampC* Streck ARM-D Kits (Streck, La Vista, NE) (102, 103).

The *ampC* Streck ARM-D Kits detect clinically relevant resistance gene families, *CMY02, DHA, ACC, EBC, FOX,* and *MOX*, and differentiate between plasmid-mediated and chromosomal AmpC β-lactamase resistance. AmpC genes mediate resistance to cephalothin, cefazolin, cefoxitin, most penicillins, and β-lactamase inhibitor-β-lactam combinations (104). The β-Lactamase Streck ARM-D Kits allow the detection of nine clinically relevant carbapenemases, ESBLs, and plasmid-mediated *ampC* gene families, with *KPC, NDM, OXA-48, IMP, VIM,* and *DHA* genes representing carbapenemases. The AmpC enzyme is the most commonly found gene (105), and *CTX-M* is the most widespread ESBL type (106). The genes *CMY-2, CTX-M-14,* and *CTX-M-15* represent ESBLs.

The genus and species of the isolates were identified, before WGS analysis, using the automated mass spectrometry microbial identification system, VITEK MS (bioMérieux, Durham, NC), which utilizes MALDI-TOF technology. Standard manufacturer laboratory procedures and policies were implemented for the VITEK MS instrument. Currently, most veterinary diagnostic laboratories rely on accurately identifying *Enterobacterales* and specifically *E. coli* using phenotypic-based approaches such as lab plate identification and MALDI-TOF. However, the training of personnel, quality, and reproducibility of phenotypic methods vary by manufacturer, which may result in incorrect interpretation (107, 108). MALDI-TOF identifies bacterial species by comparing the peptide mass fingerprint (PMF) of the query organism with the PMFs contained in the database. While MALDI-TOF is gradually replacing conventional methods for microorganism identification in clinical laboratories, the inherent similarities among organisms and the limited number of PMFs in the database can result in poor species differentiation and misidentifications (109).

Susceptibility test via disc diffusion was performed for 17 antibiotics (amikacin, ampicillin, aztreonam, cefazolin, cefepime, cefoxitin, ceftazidime, ceftriaxone, ertapenem, gentamicin, levofloxacin, meropenem, piperacillin/tazobactam, tetracycline, tigecycline, tobramycin, and trimethoprim/sulfa), following Clinical and Laboratory Standards Institute guidelines (110).

### Whole-genome sequencing

Bacterial isolates resistant to at least one drug in *two or more* antimicrobial classes (22 samples) were sent to the Athens Veterinary Diagnostic Laboratory at the University of Georgia (Athens, GA, US) for WGS. DNA was extracted from overnight cultures in BHI broth using the DNeasy UltraClean Microbial Kit (QIAGEN, https://www.qiagen.com/in) following the manufacturer’s instructions. DNA samples were barcoded, and sequencing libraries were prepared using the Nextera DNA Flex Library Prep kit (Illumina). Sequencing was performed using MiSeq 2 × 150 bp chemistry (Illumina) (111).

### Bioinformatic analysis

Paired-end reads were qualitatively checked using FastQC v0.11.9 (http://www.bioinformatics.babraham.ac.uk/projects/fastqc/) in GalaxyTrakr. Raw reads were trimmed and quality-filtered (Phred score >30) using Trimmomatic v0.38 (112). *De novo* assemblies were produced from the quality-filtered paired reads with SPAdes v3.12.0 (113). Assembly metrics were evaluated using the Quality Assessment Tool for Genome Assemblies v5.2.0 (114). Sequences were deposited in GenBank under BioProject PRJNA1147276. All samples were further analyzed using the Average Nucleotide Identity method. Genomes were annotated with PROKKA v1.14.5-gompi-2022a (115). The ST of the isolates was determined using MLST v.2.22.0 (116) in the GalaxyTrakr bioinformatic platform (117). Antimicrobial resistance gene presence was identified with ResFinder 4.0 (118), and the presence of plasmids was also identified by PlasmidFinder (119), also in the GalaxyTrakr platform. Plasmids carrying resistance genes shared between humans and companion animals were evaluated and illustrated as a graphical map using Proksee (120). All plasmids underwent a nucleotide query against their corresponding isolates to determine whether the resistance genes were located on the chromosome or plasmid using NCBI BLAST + blastn (Galaxy Version 2.14.1 + galaxy2) (121–123). Parameters chosen included one hit and a 90% cutoff. Core-genome phylogenetic analysis was completed with PARsnp from the Harvest Package (124). Trees were visualized in FigTree v1.4.4 (http://tree.bio.ed.ac.uk/software/figtree). Each isolate underwent ClermonTyping to assign one of the seven main *E. coli* phylogroups.

### Statistics

Microbiological results were analyzed in a cross-sectional manner at the end of all three visits. All participants who were lost to follow-up or early termination were excluded from the analysis. Fisher’s exact test of independence was used to determine an association between the outcome (colonization of MDR-GNB bacteria in subjects) and the exposure (colonization of MDR-GNB bacteria in pets) at the end of the study. Specifically, the study set out to determine whether there is an association between pets that were colonized with MDR-GNB bacteria during the specified period and pet owners who were colonized with carriers of MDR-GNB bacteria during the study period. For all tests, *P*-value < 0.05 indicated statistical significance. Data management and Fisher’s exact test of independence were performed using R v3.6.2 https://www.rdocumentation.org/packages/stats/versions/3.6.2, and RStudio v1.2.5033 software, https://posit.co/download/rstudio-desktop/.

## Data Availability

The sequencing data of this study materials have been deposited in GenBank database under BioProject accession number PRJNA1147276.

## SUPPLEMENTAL MATERIAL

Data S1 spectrum.00503-25-s0001.pdf Detailed instructions for participant regarding stool collection.

Data S2 spectrum.00503-25-s0002.tiff Phylogenetic tree showing genetic relatedness to ancestral *E. coli* and *Salmonella Typhimurium*.

Data S3 spectrum.00503-25-s0003.tiff Phylogenetic tree showing genetic relatedness to ancestral *E. coli*.

Data S4 spectrum.00503-25-s0004.xlsx Spreadsheet detailing results of WGS.

Data S5 spectrum.00503-25-s0005.xlsx Spreadsheet detailing metadata from participants and also laboratory results.

ASM does not own the copyrights to Supplemental Material that may be linked to, or accessed through, an article. The authors have granted ASM a non-exclusive, world-wide license to publish the Supplemental Material files. Please contact the corresponding author directly for reuse.
