# Supplementary material for: No evidence of multidrug-resistant Enterobacterales transmission between healthy companion animals and pet owners in the greater Atlanta area: a pilot study
Source: Microbiol Spectr. 2025 Oct 8;13(11):e00503-25. doi: 10.1128/spectrum.00503-25 (PMC12584688; doi:10.1128/spectrum.00503-25)
Supplement: Data S1 — Detailed instructions for participant regarding stool collection. [file spectrum.00503-25-s0001.pdf]

Supplementary Data 1: Stool Collection Instructions for pet owner and pets

Human participant Stool Collection instructions

1. Add date of collection to pre-supplied subject specific label and place on side of stool collection container
2. Place plastic holder under the toilet seat
3. Remove cap of stool collection container
4. Place open container within the plastic holder
5. Collect stool in container.
6. DO NOT mix urine with stool sample
7. Remove container and replace cap on container
8. Insert entire stool collection container into supplied specimen bag.
9. Wash hands thoroughly with soap and water
10. Place specimen bag into supplied transport bag
11. Insert frozen refrigerated packs* around the specimen bag within the transport bag

*Refrigerant packs should be frozen for at least 24 hours prior to use.

Companion Animal Stool Collection Instructions

1. Gloves are provided to be used during collection
2. Collect a fresh stool sample from pet
3. Ok to use any plastic bag you have available
4. Use a separate plastic bag to collect each individual pet's sample
5. For cats, it is ok to collect from litter box, provided the specimen is <24 hours old
    a. If more than one cat in household, separate cats individually and place one at a time in a room with a clean litterbox until cat has provided a specimen (to ensure you know which cat provided the stool)
    b. Collect specimen in small plastic bag
    c. It is ok if a small amount of litter is included with specimen
6. For dogs, collect specimen immediately after dog makes stool, as you would on a walk with the dog
    a. It is ok if there is a small amount of grass or other debris from collecting the stool
7. Place plastic bag with stool inside into individual specimen bag for each pet (provided)
8. Add date of collection to pre-supplied pet-specific label(s) and place on outside of specimen bag(s)
9. Place specimen bag(s) within supplied transport bag along with subject sample and refrigerant packs*

*Refrigerant packs should be frozen for at least 24 hours prior to use.

Please contact study staff with any questions regarding stool collection process.
