# Supplementary figures and images for: No evidence of multidrug-resistant Enterobacterales transmission between healthy companion animals and pet owners in the greater Atlanta area: a pilot study
Source: Microbiol Spectr. 2025 Oct 8;13(11):e00503-25. doi: 10.1128/spectrum.00503-25 (PMC12584688; doi:10.1128/spectrum.00503-25)

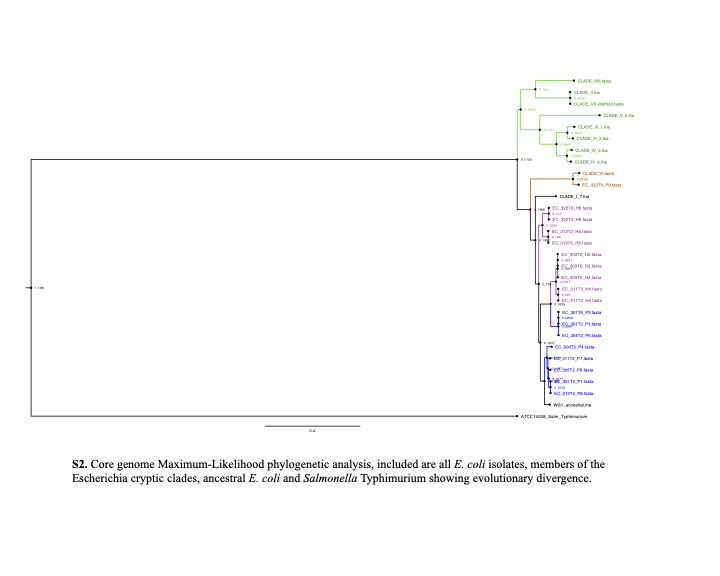

Supplement: Data S2 — Phylogenetic tree showing genetic relatedness to ancestral E. coli and Salmonella Typhimurium. [file spectrum.00503-25-s0002.tiff]

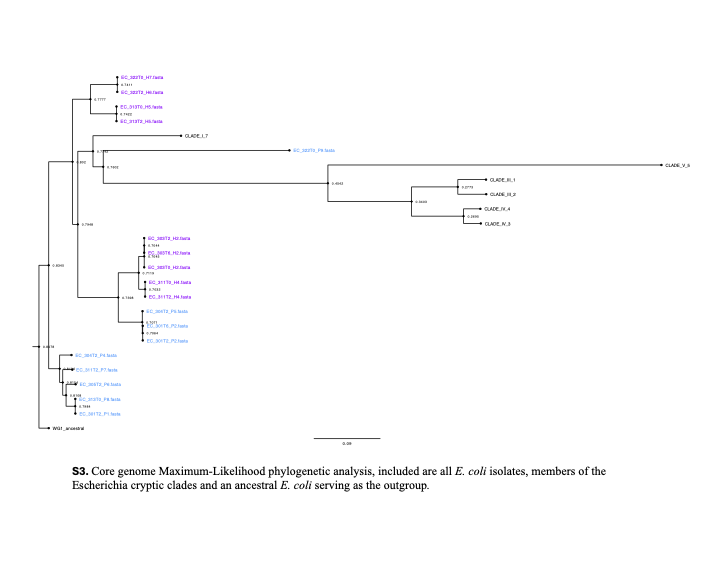

Supplement: Data S3 — Phylogenetic tree showing genetic relatedness to ancestral E. coli. [file spectrum.00503-25-s0003.tiff]
